# Sublethal and transgenerational effects of dinotefuran on biological parameters and behavioural traits of the mirid bug *Apolygus lucorum*

**DOI:** 10.1038/s41598-019-57098-z

**Published:** 2020-01-14

**Authors:** Zengbin Lu, Song Dong, Chao Li, Lili Li, Yi Yu, Xingyuan Men, Shuyan Yin

**Affiliations:** 1 Institute of Plant Protection, Shandong Academy of Agricultural Sciences, Jinan, 250100 China; 2 Maize Research Institute, Shandong Academy of Agricultural Sciences/National Engineering Laboratory of Wheat and Maize/Key Laboratory of Biology and Genetic Improvement of Maize in Northern Yellow-Huai River Plain, Ministry of Agriculture and Rural Affairs, China, Jinan, 250100 China; 3 College of Plant Protection, Shandong Agricultural University, Tai’an, 271018 China

**Keywords:** Agroecology, Agroecology, Entomology, Entomology

## Abstract

The mirid bug, *Apolygus lucorum*, has become a major pest of many crops and fruit trees since the widespread adoption of Bt cotton in northern China. Neonicotinoid insecticides, such as dinotefuran, applied to control this pest may show sublethal effects, but evidence for such effects is lacking. Here, we investigated the sublethal and transgenerational effects of dinotefuran on biological parameters and feeding behavioural traits of *A*. *lucorum* using the age-stage, two-sex life table and electrical penetration graphs (EPGs), respectively. The LC_10_ and LC_30_ of dinotefuran against 3^rd^-instar nymphs of *A*. *lucorum* were 14.72 and 62.95 mg L^−1^, respectively. These two concentrations significantly extended the development duration from 3^rd^-instar nymph to adult in parent generation (F0). LC_30_ also increased the oviposition period and male adult longevity and reduced nymphal survival rate in the F0. For offspring generation (F1), the egg duration, preadult duration, and total preoviposition period were significantly lower at LC_10_ than in the control, and the egg duration, duration of 4^th^-instar nymphs, preadult duration, oviposition period, and fecundity were also decreased at LC_30_. However, the four demographic parameters of F1 generation, namely, net reproductive rate (*R*_0_), intrinsic rate of increase (*r*), finite rate of increase (λ), and mean generation time (*T*), were not affected by dinotefuran. The significant differences in the number of probes and duration of each of four feeding waveforms failed to be detected when *A*. *lucorum* nymphs treated by dinotefuran feed on Bt cotton plants without insecticide exposure. Overall, the dinotefuran concentrations tested here have sublethal, but no transgenerational impacts on *A*. *lucorum*.

## Introduction

Mirid bugs, including *Apolygus lucorum*, have become the key pests of cotton and fruit trees owing to the major reduction in broad-spectrum insecticide use associated with the wide-scale adoption of Bt cotton in China since 1997^1,2^. The nymphs and adults of these bugs mainly feed on the young tissues of crops, including the leaves, bolls, fruits, terminal meristems, and other tissues, resulting in the stunting of plants and dropping of bolls and fruits, and in turn causing serious yield loss^3^. Broad-spectrum insecticides, such as organophosphates and pyrethroids, have been extensively applied to reduce the infestation by these insects^4–6^. However, many environmental risks are related to the use of these conventional insecticides, e.g., insect resistance^7,8^, with negative effects to natural enemies^9,10^ and on human health. Thus, it is crucial that insecticides with highly effective to pests and low-toxicity to mammals should be adopted to manage mirid bugs to reduce the environmental risks.

Neonicotinoid insecticides are among the most widely used pesticides globally, with the advantages of favourable toxicological properties, flexible use, and systemic activity^11,12^. They selectively act as agonists of insect nicotinic acetylcholine receptors (nAChRs), preventing signal transduction and in turn resulting in a lasting impairment of the nervous system and the death of insect^12,13^, and such insecticides have been used to control aphids, whiteflies and thrips in many agricultural crops^14^. In addition to kill the pests directly, these insecticides also exhibited sublethal effects on the physiological and behavioural traits of insect pests, such as survival^15,16^, development duration^17–19^, fecundity^16,18,20–22^, and feeding behaviour^23–27^. For example, Pan *et al*.^18^ observed reduction in adult longevity, oviposition period, fecundity, and egg hatching rate but an increase in the pre-oviposition period of *A*. *lucorum* at either LD_10_ or LD_40_ of cycloxaprid. Daniels *et al*.^27^ reported that a sublethal dose of thiamethoxam (0.8 mg L^−1^) reduced xylem feeding by *Rhopalosiphum padi*. Thus, the sublethal effects caused by these insecticides should be rigorously assessed to determine the response characteristics of insect pests.

Electrical penetration graphs (EPGs) are an effective tool to study the feeding behaviour of sap-sucking pests such as aphids^23–25,27^. However, phytophagous mirids produce no salivary sheath^28^ and perform macerating or lacerating behaviour^28–31^. As a consequence, the feeding behaviour of these bugs observed on the EPGs recording may be different from those of other sap-sucking pests. Fortunately, the biological meaning of the feeding waveforms in a few species of mirid bugs was recently clarified in several studies^29,32,33^. Additionally, EPGs were also applied to reveal the impacts of neonicotinoid insecticides on the feeding behaviour in *Aphis gossypii*^24,34^, *Myzus persicae*^23,35^, *Sitobion avenae*^25^, and *R*. *padi*^27,36^. Cui *et al*.^25^ found that cycloxaprid significantly increased the total length of non-probing periods and inhibited the phloem ingestion of *S. avenae*. Therefore, EPGs may have the potential to reveal the sublethal effects of insecticides on feeding behavioural traits of *A*. *lucorum*.

Dinotefuran, the third generation of neonicotinoid insecticides, was developed by Mitsui Chemicals, Inc. in 2002 and has a characteristic tetrahydro-3-furylmethyl group that differs from that of most other neonicotinoids such as imidacloprid^37^. Few studies have investigated the sublethal effects of dinotefuran on *Bemisia tabaci*^19^ and *Halyomorpha halys*^38^. However, its sublethal effects on *A*. *lucorum* have not yet been reported. Here, we first assessed the acute toxicity of dinotefuran to 3^rd^-instar nymphs of *A. lucorum*. Then, the sublethal effects of dinotefuran on the biological parameters and feeding behaviour of parent (F0) and offspring generations (F1) were determined using the age-stage, two-sex life table and EPGs, respectively. The purpose was to clarify whether the population-level performance and behavioural traits of *A. lucorum* were influenced by dinotefuran exposure.

## Results

### Acute toxicity of dinotefuran to *Apolygus lucorum*

At 48 h, the linear regression equation derived from the concentration-mortality response bioassay was *Y* = 1.2*X* − 2.683 (*χ*^2^ = 3.44, *P* = 0.33; *X* represents the log-transformed concentrations of dinotefuran, and *Y* represents the probability of *A*. *lucorum* mortality). The LC_10_, LC_30_, and LC_50_ of dinotefuran against 3^rd^-instar nymphs of *A. lucorum* were 14.72 (95% confidence interval: 2.66–33.62 mg L^−1^), 62.95 (24.95–102.47 mg L^−1^), and 172.20 mg L^−1^ (106.93–243.74 mg L^−1^), respectively.

### Sublethal effects of dinotefuran on the F0 generation of *Apolygus lucorum*

**Figure 1:**
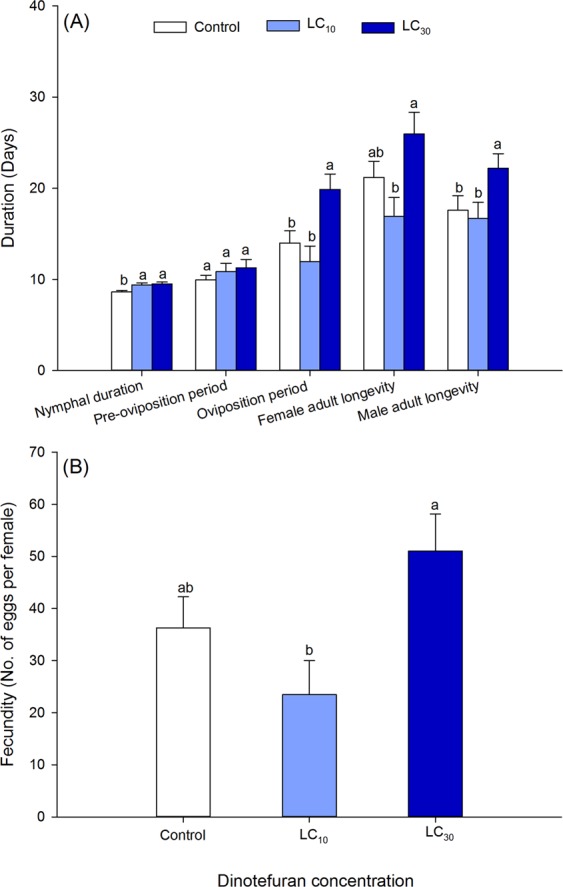
Sublethal effects of dinotefuran on development duration (**A**) and fecundity (**B**) of the F0 generation of *Apolygus lucorum*. Nymphal duration represents the development duration from 3^rd^-instar nymph to adult. Data are mean ± standard errors (SEs). The same lowercase letters within each parameter indicate that treatments are not significantly different from each other based on one-way ANOVA followed by Tukey’s multiple comparisons test at *P* ≤ 0.05.

The development duration and fecundity of F0 generation of *A*. *lucorum* are shown in Fig. 1. Compared with the control, the development duration from 3^rd^-instar nymph to adult was significantly extended by 0.78 and 0.87 days at LC_10_ and LC_30_, respectively (*F* = 6.73, df = 2, 279, *P* = 0.002). LC_30_ also significantly increased the oviposition period and male adult longevity compared with the control and the LC_10_ (oviposition period: *F* = 6.43, df = 2, 74, *P* = 0.003; male adult longevity: *F* = 3.16, df = 2, 163, *P* = 0.045). In contrast, the nymphal survival rate from 3^rd^-instar nymph to adult was reduced at LC_30_ (Control: 66.67 %; LC_10_: 64.44 %; LC_30_: 40 %; *X*^2^ = 8.04, *P* = 0.018) and the female adult longevity and fecundity were significantly lower at LC_10_ than at LC_30_ (female adult longevity: *F* = 4.60, df = 2, 105, *P* = 0.012; fecundity: *F* = 4.17, df = 2, 105, *P* = 0.018). However, the pre-oviposition period did not differ between dinotefuran treatments and the control (*F* = 0.89, df = 2, 74, *P* = 0.415).

### Sublethal effects of dinotefuran on the F1 generation of *Apolygus lucorum*

**Figure 2:**
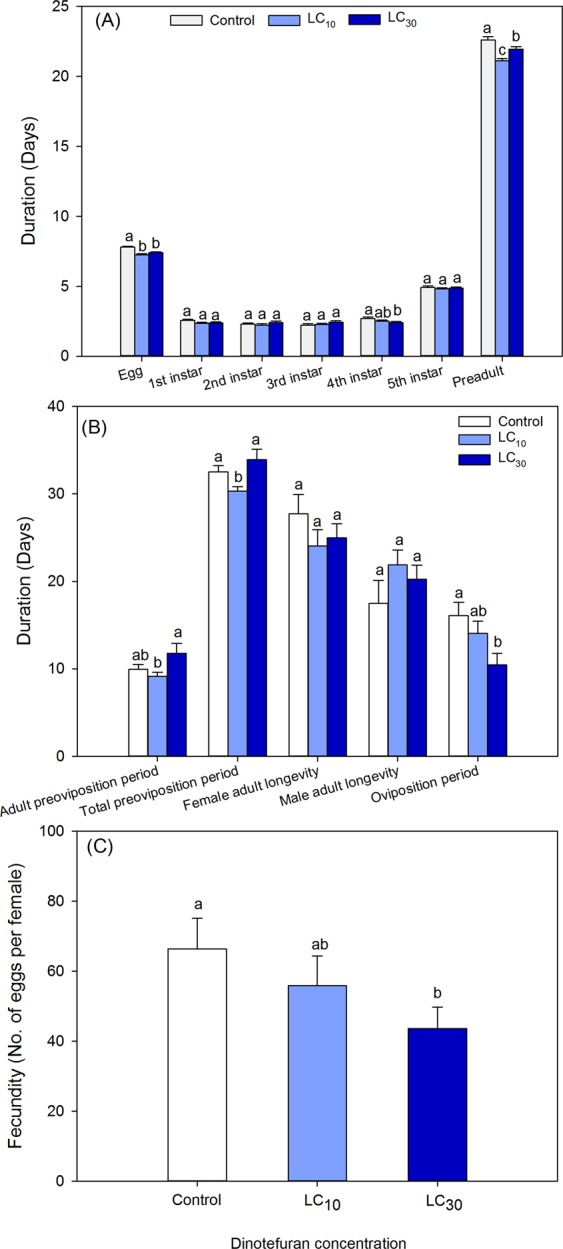
Sublethal effects of dinotefuran on development duration of immature (**A**) and adult (**B**) and fecundity (**C**) of the F1 generation of *Apolygus lucorum*. The standard errors (SEs) were estimated using bootstrap technique with 100,000 resamplings. The same lowercase letters within each parameter indicate that treatments are not significantly different from each other based on a paired bootstrap test at *P* ≤ 0.05.

The development duration and fecundity of F1 generation of *A*. *lucorum* are shown in Fig. 2. Both LC_10_ and LC_30_ significantly shortened the egg duration and preadult duration. Compared with the control, the duration of 4^th^-instar nymph, oviposition period, and female fecundity were markedly decreased by LC_30_, and the total preoviposition period was also reduced by LC_10_. However, other biological parameters including the preadult survival rate (Control: 43 %; LC_10_: 50 %; LC_30_: 47 %) and the four demographic parameters, namely, *R*_0_, *r*, λ, and *T*, were not affected by these two concentrations (Table 1).

**Table 1:** Sublethal effects of dinotefuran on demographic parameters of the F1 generation of *Apolygus lucorum.*

Parameter	Control	LC_10_	LC_30_
Net reproductive rate, *R*_0_	16.47 ± 3.39a	11.92 ± 2.69a	10.10 ± 2.11a
Intrinsic rate of increase, *r* (d^−1^)	0.07 ± 0.01a	0.06 ± 0.01a	0.06 ± 0.01a
Finite rate of increase, *λ* (d^−1^)	1.07 ± 0.01a	1.06 ± 0.01a	1.06 ± 0.01a
Mean generation time, *T* (d)	40.96 ± 0.92a	39.00 ± 0.88a	39.37 ± 0.90a

The standard errors (SEs) were estimated using bootstrap technique with 100,000 resamplings. The same lowercase letters within the same row indicate that treatments are not significantly different from each other based on a paired bootstrap test at* P* ≤ 0.05.

**Figure 3:**
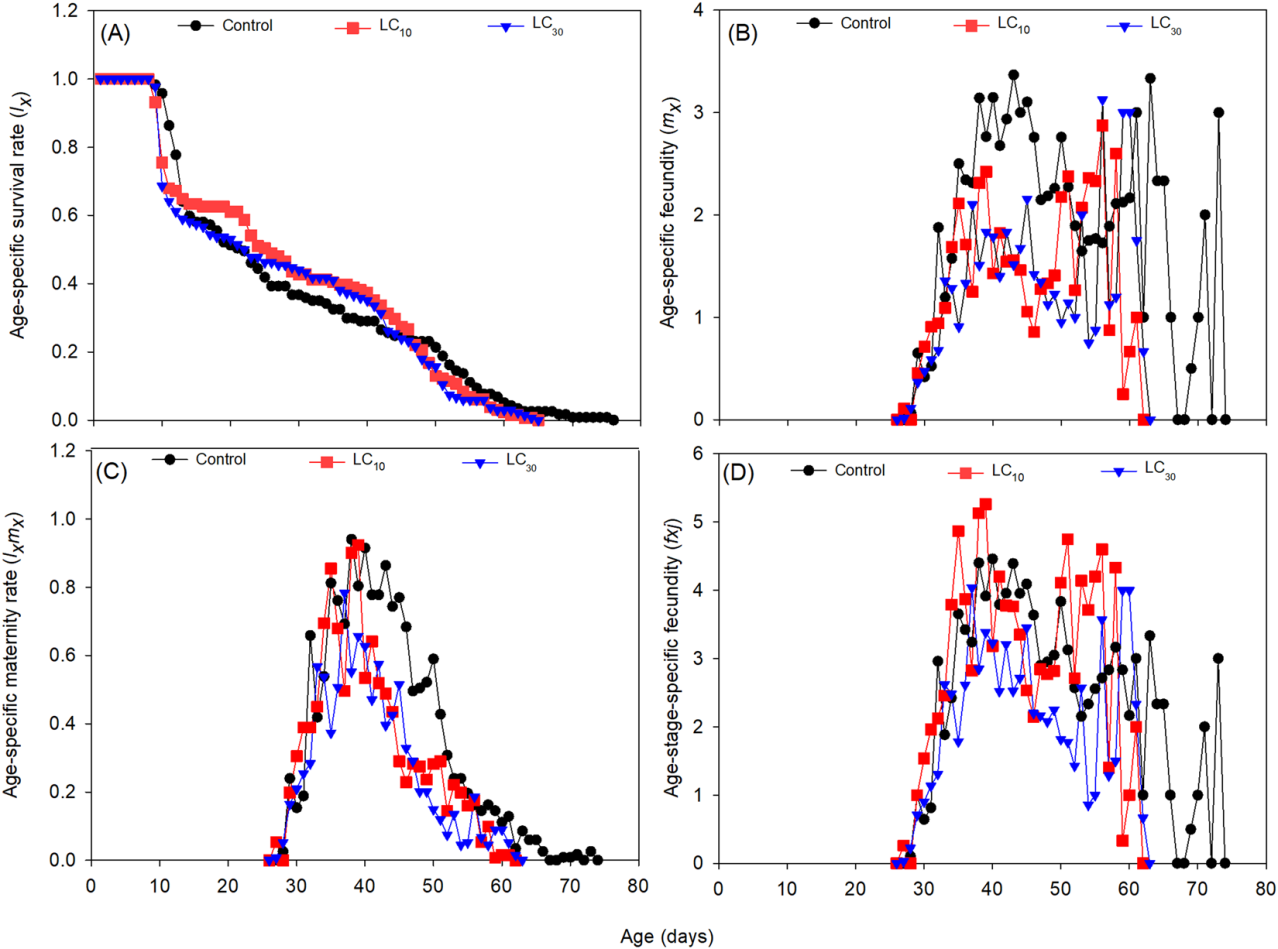
Sublethal effects of dinotefuran on the age-specific survival rate (*l*_*x*_, **A**), age-specific fecundity (*m*_*x*_, **B**), age-specific maternity (*l*_*x*_*m*_*x*_, **C**), and age-stage specific fecundity (*f*_*xj*_, **D**) of the F1 generation of *Apolygus lucorum*.

The sublethal effects of dinotefuran on the age-specific survival rate (*l*_*x*_), age-specific fecundity (*m*_*x*_), age-specific maternity rate (*l*_*x*_*m*_*x*_), and age-stage-specific fecundity (*f*_*xj*_) of *A*. *lucorum* are shown in Fig. 3. The *l*_*x*_ gradually decreased with ages and the maximum ages reached 76, 65, and 65 days in the control, LC_10_, and LC_30_, respectively. The time spans of female oviposition in the control were longer than that at the LC_10_ and LC_30_ (Control: 48 days, LC_10_: 37 days, LC_30_: 38 days). The *m*_x_ has two peaks in each treatment across whole ages, and the *f*_*xj*_ also has two peaks at LC_10_ and LC_30_, but not in the control. The highest values of *l*_*x*_*m*_*x*_ were 0.94, 0.92, and 0.78 for the control, LC_10_ and LC_30_, respectively.

### Sublethal effects of dinotefuran on the feeding behaviour of *Apolygus lucorum*

Four main electrical waveforms were identified in *A*. *lucorum* fed on Bt cotton plants: stylet probing (P), stylet insertion into cells (I), cell rupturing and salivation (B), and feeding on the cell mixture (S). Neither LC_10_ nor LC_30_ significantly affected the number of probes or the duration of each waveform (P, I, B, and S) (Table 2).

**Table 2:** Sublethal effects of dinotefuran on probing number and probe duration of *Apolygus lucorum* fed on Bt cotton plants for 6 h.

Parameter	Treatment			Statistics	
	Control	LC_10_	LC_30_	*F*_2,57_	*P*
No. of probes	6.78 ± 1.15a	8.65 ± 0.93a	8.15 ± 0.85a	0.95	0.394
P waveform (min)	8.21 ± 1.43a	8.92 ± 1.37a	5.95 ± 0.74a	1.69	0.194
I waveform (min)	15.03 ± 5.64a	13.06 ± 1.50a	12.74 ± 1.64a	0.14	0.873
B waveform (min)	17.26 ± 3.09a	16.39 ± 3.56a	17.96 ± 4.36a	0.05	0.096
S waveform (min)	13.97 ± 2.29a	20.39 ± 3.17a	18.71 ± 3.93a	0.99	0.377

P waveform represents stylet probing; I waveform represents stylet insertion into cells; B waveform represents cell rupturing and salivation; and S waveform represents feeding on the cell mixture.

## Discussion

In this study, we provided the evidence that both LC_10_ and LC_30_ of dinotefuran have no transgenerational effects on *A. lucorum* with respect to the demographic parameters: *R*_0_, *r*, λ, and *T* (Table 1). This finding was in accord with the previous reports on *A*. *gossypii* exposed to LC_20_ of cycloxaprid^39^ and *B*. *tabaci* treated by LC_25_ of imidacloprid^40^. However, significant impacts on the demographic parameters were shown in *A*. *lucorum* treated with LD_15_ of sulfoxaflor^41^; in *M*. *persicae* exposed to imidacloprid^17,23^ or thiamethoxam^20^; and in *A. gossypii*^24^, *Aphis glycines*^42^ and *R*. *padi*^43^ exposed to imidacloprid. Zhen *et al*.^41^ showed that the LD_15_ of sulfoxaflor significantly reduced the *r*, *λ*, *T*, *R*_0_, and gross reproduction rate (GRR) of the F1 generation of *A. lucorum* compared with the control. Thus, the sublethal effects of insecticides on the demographic parameters were affected by multiple factors, e.g., insect species and kinds and amount of insecticides. Since the demographic parameters reflect the performance of insect pests at the population level, the present finding indicates that dinotefuran would not affect the performance of *A. lucorum* population.

The dinotefuran concentrations tested here also significantly increased the nymphal development duration, oviposition period, and male adult longevity in F0 generation of *A*. *lucorum*, while decreased the egg duration, preadult duration, total preoviposition period and oviposition period in F1 generation (Figs. 1 and 2). Interestingly, the oviposition period of *A. lucorum* exposed to LC_30_ of dinotefuran in the F0 and F1 generation was inverse. Compensatory effects might exist in *A. lucorum* to synchronize the developmental rate of various populations. Similarly, Li *et al*.^43^ observed a longer oviposition period in *R*. *padi* treated by imidacloprid, while a shorter oviposition period was documented in *A*. *lucorum*^18,41^ and *A. glycines*^42^. Additionally, imidacloprid significantly extended the nymphal development duration of *M. persicae*^17,23^ and *R. padi*^43^. In contrast, a reduction in nymphal development duration was reported in *A*. *lucorum*^41^, *B. tabaci*^15^, *A*. *gossypii*^24,34^, and *A*. *glycines*^42^. And the reduction in preadult duration may attribute to the lower egg duration at both LC_10_ and LC_30_ of dinotefuran in F1 generation of *A*. *lucorum* (Fig. 2). The development changes in these stages might be caused by two reasons. First, the antifeedant effects of these insecticides at low concentrations^22^ negatively affected the nutrition absorption of exposed insects^22,24^. The other may be related to the disruption of hormone balance^42^.

We also showed that the nymphal survival rate in F0 generation and fecundity in F1 generation of *A*. *lucorum* were decreased at LC_30_. Many insect species also have lower survival rates in their immature stages, including *A*. *lucorum*^41^, *R. padi*^43^, *B*. *tabaci*^15,44^, *Euschistus heros*^16^, and *A*. *gossypii*^45^. Additionally, the reduction in fecundity were also observed in *A*. *lucorum* with LD_40_ of cycloxaprid^18^, *A*. *gossypii* exposed to LC_10_ and LC_40_ of cycloxaprid^34^, *A*. *glycine* with 0.20 mg L^−1^ of imidacloprid^42^, and *N*. *lugens* treated with imidacloprid and dinotefuran^46^. This phenomenon could attribute to the reduction in vitellogenin (*Vg*) and the expression level of *Vg* mRNA significantly decreased by 43.8% in F1 generation of *A*. *lucorum* whose parents were treated with LD_15_ of sulfoxaflor^41^.

EPGs analysis demonstrated that the feeding behaviour of *A. lucorum* did not differ between dinotefuran treatments and the control, indicating that these two concentrations will not increase crop injury when this pest moves to other plants. In contrast, many studies have reported negative effects of insecticides on the feeding behaviour of *A*. *gossypii*^24,34^, *M. persicae*^23,35^, *S*. *avenae*^25^, and *R*. *padi*^27,36^. Cira *et al*.^26^ found that *H*. *halys* adults that survived from sulfoxaflor exposure produced significantly fewer feeding sites than those in the control. Indeed, *A. lucorum* performs a macerating or lacerating behaviour, which is different from other sap-feeding pests such as aphids and leafhoppers^28–31^, and has a shorter ingestion duration like *Adelphocoris suturalis* in plants^47^. The different feeding strategy may led to their different responses towards insecticides. Nevertheless, further experiments are needed to clarify the relationships between the amount of insecticide used and the feeding response of *A. lucorum*.

In summary, the dinotefuran concentrations tested here showed sublethal effects, but no transgenerational effects on *A. lucorum*. It implies that dinotefuran would not increase the population size of *A. lucorum*.

## Materials and Methods

### Ethics statement

Permission was not required for insect collection, because none of the species used in the study were endangered or protected.

#### Insects

Overwintering eggs of *A. lucorum* used in this study were originally collected from a winter jujube orchard in Binzhou, Shandong Province, China, in 2016. After eggs hatched, they were reared on green bean (*Phaseolus vulgaris*) without exposure to any insecticide in transparent glass jars (10 cm in diameter, 15 cm in height). These jars were maintained in a climate-controlled chamber with a temperature of 25 ± 1 °C, relative humidity (RH) of 65 ± 5%, and photoperiod of L16: D8.

#### Insecticides

Dinotefuran (95.57% purity) was purchased from Suzhou Aotelai Chemical Group (Suzhou, Jiangsu Province, China) and used in all the following experiments.

#### Acute toxicity of dinotefuran to *Apolygus lucorum*

The acute toxicity of dinotefuran to 3^rd^-instar nymphs of *A. lucorum* was assessed using the leaf-dipping method. The stock solution of dinotefuran prepared in acetone was diluted into a series of concentrations: 68.75, 137.5, 275, 550, and 1100 mg L^−1^, using distilled water containing 1‰ (v/v) Tween-80. Distilled water containing 1‰ Tween-80 was used as the control. Fresh green beans with the same size were cut into 2-cm-long sections, dipped into each insecticide solution and the control for 20 min, and air-dried at room temperature for 2 h. These beans were then placed into the transparent plastic containers (6 cm in diameter, 7 cm in height), each of which included three 2-cm-long sections of green bean. After 5 h of starvation, 3^rd^-instar nymphs of *A. lucorum* were transferred into these containers. Each treatment was repeated three times with 15 nymphs per replicate. All the containers were then maintained in a climate-controlled chamber with a temperature of 25 ± 1 °C, RH of 65 ± 5%, and photoperiod of L16: D8. After 48 h, the nymphal mortality was recorded, and the nymphs that did not move when touched with a thin brush were regarded as dead.

#### Sublethal effects of dinotefuran on the F0 generation of *Apolygus lucorum*

The LC_10_ (14.72 mg L^−1^) and LC_30_ (62.95 mg L^−1^) were used to evaluate the sublethal effects of dinotefuran on *A*. *lucorum*. These two concentrations were prepared as the method described in the “Acute toxicity of dinotefuran to *Apolygus lucorum*” section above. Distilled water containing 1‰ Tween-80 was used as the control. There were 154, 135, and 225 of 3^rd^-instar nymphs used for the control, LC_10_, and LC_30_, respectively. After 48 h, the survivors of each treatment were individually transferred to the smaller transparent plastic container (1.5 cm in diameter, 2 cm in height) with one 1.5-cm-long green bean section free from insecticide. The development and survival of nymphs were recorded daily. After adults emerged, they were paired (1 male and 1 female) in new transparent plastic containers (1.5 cm in diameter, 2 cm in height) with one 1.5-cm-long section of green bean (as food and oviposition substrate). The old green beans were replaced by new ones daily. The eggs in the old green beans were checked and counted under a stereomicroscope until adult death. If the male adult died during the experiment, it was removed and replaced by a new one from the same treatment. All the experiments were conducted in a climate-controlled chamber with a temperature of 25 ± 1 °C, RH of 65 ± 5%, and photoperiod of L16: D8.

#### Sublethal effects of dinotefuran on the F1 generation of *Apolygus lucorum*

When the eggs laid by female adults of the F0 generation peaked, there were 117, 131, and 134 eggs randomly selected and assigned to the control, LC_10_, and LC_30_, respectively, to initiate the life table study of the F1 generation. The hatched eggs were recorded daily and the newly born nymphs were individually transferred into a new transparent plastic container (1.5 cm in diameter, 2 cm in height) with one 1.5-cm-long green bean section without exposure to any insecticide. The nymphal stage and survival were checked and recorded daily. Within 24 h of adult emergence, adults were paired (1 male and 1 female) in a transparent plastic container with one 1.5-cm-long section of green bean (as food and oviposition substrate). The old green beans were replaced by new ones daily. The eggs in the old green beans were checked and counted under a stereomicroscope until adult death. If the male adult died during the experiment, it was removed and replaced by a new one from the same treatment. All the experiments were conducted in a climate-controlled chamber with a temperature of 25 ± 1 °C, RH of 65 ± 5%, and photoperiod of L16: D8.

#### Sublethal effects of dinotefuran on the feeding behaviour of *Apolygus lucorum*

The feeding behaviour of *A. lucorum* exposed to dinotefuran on Bt cotton plants at the seedling (variety: Lumianyan 36, developed by Cotton Research Center, Shandong Academy of Agricultural Sciences, Jinan, China) were recorded using a Giga-4 direct-current electrical penetration graph system (DC-EPG, manufactured by WF Tjallingii, Wageningen University, Wageningen, Netherlands). Briefly, the 3^rd^-instar nymphs reared on green beans were exposed to LC_10_ and LC_30_ of dinotefuran and the control prepared as described in the “Acute toxicity of dinotefuran to *Apolygus lucorum*” section above for 48 h. After 5 h starvation, these nymphs were immobilized on an ice plate and then secured on a vacuum device for the attachment of wires. A gold wire (2 cm in length, 20 μm in diameter) was attached to the pronotum of individual nymph using silver glue under a stereomicroscope. The gold wire allowed the nymphs to move relatively unaffectedly with a radius equal to the length of the wire tether. Then, each nymph was connected to the amplifier before being placed on a cotton leaf. Another copper electrode was inserted into the soil in the pot with one Bt cotton plant. Finally, the entire experimental arena was covered by a Faraday cage to shield external noise and other interference. Recordings were made simultaneously on four individual Bt cotton plants over 6 h using ANA 34 software (Wageningen, The Netherlands). Nymphs and Bt cotton plants were used only once and then discarded. For each treatment, at least 20 nymphs were successfully tested. Electrical signals were identified and characterized based on the descriptions by Song *et al*.^33^ and Zhao *et al*.^32^.

#### Data analysis

The LC_10_, LC_30_, and LC_50_ values were determined based on the probit analysis. The development and fecundity of F0 generation and feeding behaviour were analysed using one-way analysis of variance (ANOVA) followed by Tukey’s multiple-range test. The nymphal survival rate of F0 generation were compared by Chi-squared test (*χ2*). All these analyses were conducted in SPSS 19.0 software (IBM Inc., New York, USA).

The life table data for all *A*. *lucorum* individuals in the F1 generation were analysed using TWOSEX-MSChart computer program^48^ according to the age-stage, two-sex life table theory^49,50^. The demographic parameters, namely, the net reproductive rate (*R*_0_), intrinsic rate of increase (*r*), finite rate of increase (λ), and mean generation time (*T*), of the F1 generation were calculated based on the following Eqs. (1–4) via the computer program. The age-specific survival rate (*l*_*x*_), the age-specific fecundity (*m*_*x*_), age-specific maternity rate (*l*_*x*_*m*_*x*_), and the age-stage-specific fecundity (*f*_*xj*_, where *x* is the age and *j* is the stage) were also obtained from the computer program.

1 $${R}_{0}=\mathop{\sum }\limits_{x=0}^{\infty }\,{l}_{x}{m}_{x}$$

2 $$\mathop{\sum }\limits_{x}^{\infty }\,{e}^{-r(x+1)}{l}_{x}{m}_{x}=1$$

3 $${\rm{\lambda }}={e}^{r}$$

4 $$T=\frac{\mathrm{ln}\,{R}_{0}}{r}$$

The means and standard errors of all the life history traits and demographic parameters were estimated using bootstrap technique with 100,000 resamplings^51,52^, and the differences among treatments were compared using the paired bootstrap test based on the confidence intervals^53^. All the figures were created in SigmaPlot 14.0 software (Systat Software Inc., San Jose, CA). For all the test, *α* = 0.05.
